# Impact of individualized target mean arterial pressure for septic shock resuscitation on the incidence of acute kidney injury: a retrospective cohort study

**DOI:** 10.1186/s13613-018-0468-5

**Published:** 2018-12-10

**Authors:** Rajat N. Moman, Stuart A. Ostby, Abbasali Akhoundi, Rahul Kashyap, Kianoush Kashani

**Affiliations:** 1 Multidisciplinary Epidemiology and Translational Research in Intensive Care (METRIC), Division of Pulmonary and Critical Care Medicine, Department of Medicine, Mayo Clinic, 200 First Street SW, Rochester, MN 55905 USA; 2 Department of Anesthesiology and Perioperative Medicine, Mayo Clinic, Rochester, MN USA; 3 Department of Obstetrics and Gynecology, University of Alabama Birmingham, Birmingham, AL USA; 4 Division of Pulmonary and Critical Care Medicine, Department of Medicine, Mayo Clinic, Rochester, MN USA; 5 Anesthesia Clinical Research Unit, Department of Anesthesiology and Perioperative Medicine, Mayo Clinic, Rochester, MN USA; 6 Division of Nephrology and Hypertension, Department of Medicine, Mayo Clinic, Rochester, MN USA

**Keywords:** Blood pressure target, Early goal-directed therapy, Fluid resuscitation, Hypertension, Severe sepsis

## Abstract

**Background:**

To examine the relationship between delta mean arterial pressure (ΔMAP; MAP change between pre-admission minus post-resuscitation) and acute kidney injury (AKI) among patients with septic shock. In this retrospective, single-center cohort study of adult patients pre-admission MAP is defined as the median MAP recorded from 365 to 7 days before admission. Post-resuscitation MAP was median MAP during the 7th hour after initiating resuscitation.

**Results:**

In our cohort (*N* = 233; 55% male), the median (interquartile range [IQR]) age was 71 (58–81) years and the median (IQR) acute physiology, age, chronic health evaluation (APACHE) III score was 81 (66–97). Although those in the lowest ΔMAP quartile (−24.5 to 3.9 mmHg) had no demographic differences compared with the rest of the cohort, the odds ratio for AKI was 0.26 (95% CI 0.11–0.57) after adjustment for other known AKI risk factors. Among patients with a history of hypertension, the lowest quartile had an odds ratio for AKI of 0.12 (95% CI 0.04–0.37) after adjusting for risk factors for AKI in this cohort.

**Conclusions:**

The incidence of AKI was lowest among those whose post-resuscitation MAP was closest to or higher than their pre-admission MAP. Further study regarding the effect of targeting the pre-admission MAP for post-resuscitation on the incidence of AKI is warranted.

**Electronic supplementary material:**

The online version of this article (10.1186/s13613-018-0468-5) contains supplementary material, which is available to authorized users.

## Background

Acute kidney injury (AKI) is a devastating sequela of critical illnesses [1]. Sepsis is a common pathway to AKI. The Surviving Sepsis Campaign Guidelines [2] recommend a mean arterial pressure (MAP) of 65 mmHg or higher as the goal of resuscitation (Grade 1C recommendation) to minimize the risk of death and end-organ failure. However, this recommendation is not supported by substantial evidence, and whether a goal MAP of 65 mmHg is adequate for all patients remains controversial [3, 4].

Sepsis-associated AKI (SA-AKI) is a common and clinically significant condition. Sepsis is associated with AKI in 42–48% of cases in the intensive care unit (ICU) [1, 5, 6]. Compared with other non-sepsis causes of AKI, SA-AKI is associated with higher ICU and in-hospital mortality rates [5]. Patients with severe sepsis and AKI had higher 90-day mortality rates than those with severe sepsis alone [7]. Patients with even a modest increase in serum creatinine (sCR) have markedly higher health care costs, hospital length of stay, and risk of death [8]. A critical need exists to determine optimal resuscitation strategies that prevent SA-AKI or its progression in patients with septic shock.

In a recent investigation, Asfar et al. [4] randomized patients with septic shock to high (80–85 mmHg) or low (65–70 mmHg) MAP targets and observed no difference in 28-day mortality rates between groups. A subset of 167 patients with chronic arterial hypertension had a lower incidence of sCR doubling and required less renal replacement therapy when randomized to the high MAP target group. However, the higher MAP target group also had a higher incidence of atrial fibrillation. Chronic arterial hypertension results in a rightward shift of the autoregulatory pressure-organ perfusion curve [9, 10]. Therefore, an increased MAP target may improve organ perfusion for patients with higher pre-admission MAP [3, 4, 11]. Given the results of previous studies, the pre-admission blood pressure of patients in septic shock may need to be considered when defining an appropriate MAP goal for optimal resuscitation [3, 4, 11–13].

Appropriate MAP targets for resuscitation are controversial, and previous studies suggest that the pre-admission blood pressure may help determine the optimal resuscitation MAP target. To date, no published studies have assessed whether patients would benefit from a specific post-resuscitation MAP target that is similar to or higher than the pre-admission MAP. We conducted a retrospective cohort study of patients who were treated for severe sepsis and septic shock. We examined the association between the achieved MAP target and the pre-admission MAP, and AKI incidence was the primary outcome.

## Methods

### Approval of study design

This retrospective cohort study was reviewed and approved by the Mayo Clinic Institutional Review Board (protocol number 14-002109). Informed consent was waived for patients who provided research authorization.

### Participants

We conducted a study of adult patients (≥ 18 years old) who received care at a tertiary care academic hospital for severe sepsis or septic shock. Consecutive adult patients admitted to the Medical ICU from January 2007 through January 2009 were included in the study if their records included a history of noninvasive blood pressure monitoring. We excluded patients who developed AKI before sepsis and those with end-stage renal disease or receiving hemodialysis before sepsis. We also excluded patients who never had urine output measured with a Foley catheter, had a ureteral stent, were in the ICU for fewer than 6 h, or did not have sufficient clinical information available. In order to evaluate the impact ΔMAP on the AKI incidence, we excluded patients who met AKI criteria by oliguria during first 6 h of resuscitation.

### Data collection

Patient demographics, pre-admission and post-resuscitation MAP, body mass index (BMI), baseline sCR from 6 months to 7 days prior to hospitalization, sCR during hospitalization, use of inotropic and vasoactive agents, fluid balance, preexisting conditions, and urine output were abstracted from the electronic health record. Charlson comorbidity index (CCI) [14], sequential organ failure assessment (SOFA) score [15], and acute physiology, age, chronic health evaluation (APACHE) III [16] scores at 24 h were calculated.

### Study definitions

The pre-admission MAP was defined as the median of all MAPs recorded from 365 to 7 days before ICU admission. We chose the median because it was less likely to be skewed by an outlier MAP measurement compared to a mean value. By comparing the median and mean pre-admission MAPs of the cohort, we found the mean pre-admission MAP had a mean of 82.2 mmHg and standard deviation of 10.5 mmHg; the median pre-admission MAP had a mean of 81.5 mmHg and standard deviation of 10.8 mmHg (*R*^2^ 0.97, *p* < .0001). Post-resuscitation MAP was defined as the median of all MAPs during the 7th hour after initiation of sepsis resuscitation. This definition of post-resuscitation MAP was chosen because the goal of resuscitation is to have a stable blood pressure at the end of 6 h of treatment. The ΔMAP, as an independent variable, was defined as the pre-admission MAP minus the post-resuscitation MAP. Therefore, if the value of a patient’s post-resuscitation MAP is higher than their pre-admission MAP, the ΔMAP would be negative. Conversely, if the value of their post-resuscitation MAP is lower than their pre-admission MAP, the ΔMAP would be positive. All MAPs before admission were determined from noninvasive blood pressure measures. The primary outcome, AKI, was defined by Kidney Disease Improving Global Outcomes criteria [17]. sCR and urine output (UOP) were used where: stage 1 was defined as sCR 1.5–1.9 times baseline or greater than or equal to 0.3 mg/dl increase, UOP < 0.5 cc/kg/h for 6–12 h; stage 2 defined as sCR 2.0–2.9 times baseline, UOP < 0.5 cc/kg/h for greater than or equal to 12 h; and stage 3 defined as sCR 3.0 times baseline or increase to greater than or equal to 4.0 or initiation of renal replacement therapy, UOP < 0.3 cc/kg/h for greater than or equal to 24 h or anuria for greater than or equal to 12 h.

### Statistical analysis

Demographic data are shown as frequency count and percentage for categorical variables and median and interquartile range (IQR) for continuous variables. Categorical variables were analyzed with the *χ*^2^ test. ΔMAP was examined as a continuous variable and also by quartiles as a categorical variable, with comparisons made between the ΔMAP quartiles. Backward stepwise logistic regression analyses were performed. Odds ratios (ORs) and c-statistics were calculated. All *p* values < .05 in a 2-sided hypothesis were considered statistically significant. Statistical analyses were conducted with JMP software (version 10.0.0; SAS Institute Inc).

## Results

**Fig. 1:**
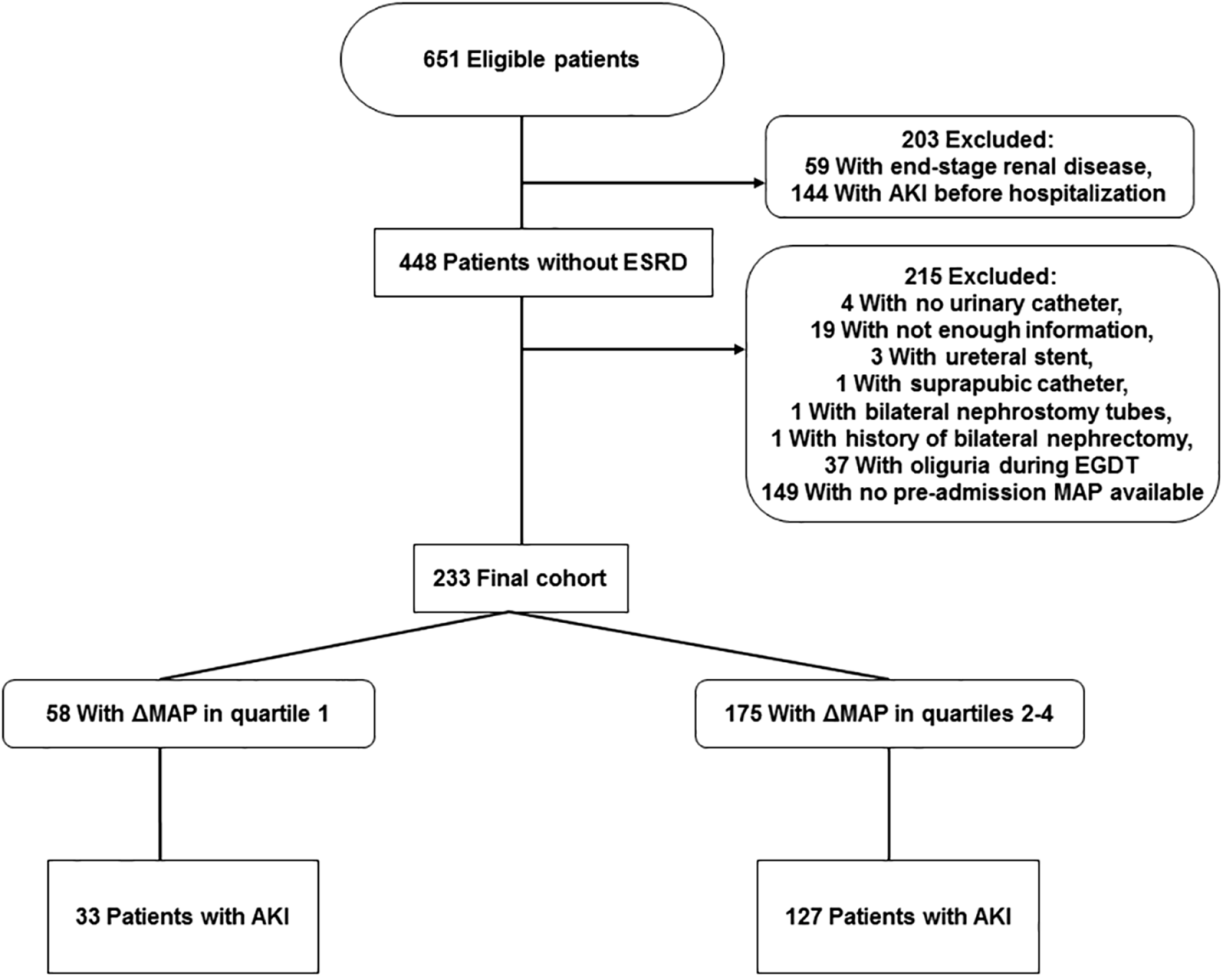
Flow diagram. The chart shows patient inclusion and exclusion in the study. AKI indicates acute kidney injury; EGDT, early goal-directed therapy; ESRD, end-stage renal disease; MAP, mean arterial pressure; ΔMAP, pre-admission MAP minus the post-resuscitation

We identified 651 patients with severe sepsis or septic shock during the study period. After exclusions, 233 patients were included in the final analysis (Fig. 1); the median (IQR) age was 71 (58–81) years, 55% of patients were male, and the median (IQR) APACHE III score was 81 (66–97). One hundred sixty patients (69%) had AKI developed during treatment for severe sepsis or septic shock. Table 1 shows patient characteristics, stratified by presence or absence of AKI.

**Table 1:** Patient characteristics, stratified by acute kidney injury status (*N* = 233)

Patient characteristics	No AKI (*n* = 73)	AKI (*n* = 160)	*p* value
Age, median (IQR), year	66.8 (56.0–80.8)	72.8 (59.7–81.6)	.21
Body mass index, median (IQR), kg/m^2^	25.2 (21.8–28.6)	27.9 (23.2–33.1)	.01
Male sex, No. (%)	45 (61.6)	83 (51.9)	.20
Pre-admission MAP, median (IQR), mmHg^a^	83.8 (76.0–91.5)	78.5 (73.4–87.6)	.05
MAP at first hour of admission, median (IQR), mmHg	67.5 (59.0–78.0)	65.0 (57.0–73.3)	.1
MAP at first hour of resuscitation, median (IQR), mmHg	62.3 (57.0–73.3)	63.0 (55.8–70.9)	.2
Post-resuscitation MAP, median (IQR), mmHg^b^	71.0 (66.0–79.0)	66.0 (61.0–73.0)	.01
ΔMAP from Baseline to 7th hour of resuscitation, median (IQR), mmHg^c^	11.0 (0.5–20.3)	12.4 (5.7–19.8)	.31
Charlson comorbidity index, median (IQR)	6 (4–8.5)	7 (5–11)	.02
APACHE III score, 24 h, median (IQR)	67 (55.5–79)	88 (72–103)	.01
SOFA score, day 1, median (IQR)	5 (3–7)	7 (5–10.8)	.01
Culture positive septic shock (%)	31 (42)	85 (53)	.2
Positive culture source			.7
Blood (%)	7 (10)	20 (13)	
Urine (%)	5 (7)	19 (12)	
Respiratory (%)	16 (22)	33 (20)	
Wound (%)	1 (1)	6 (4)	
Other (%)	2 (2)	7 (4)	
Culture negative septic shock (%)	42 (58)	75 (47)	.2
Preexisting condition, No. (%)			
Hypertension	38 (52.1)	115 (71.9)	.01
Myocardial infarction	10 (13.7)	39 (24.4)	.08
Congestive heart failure	8 (11.0)	37 (23.1)	.03
Peripheral vascular disease	3 (4.1)	25 (15.6)	.02
Dementia	5 (6.8)	7 (4.4)	.52
Cerebrovascular accident	6 (8.2)	34 (21.3)	.01
Chronic pulmonary disease	16 (21.9)	54 (33.8)	.09
Rheumatic heart disease	8 (11.0)	15 (9.4)	.81
Diabetes mellitus	15 (20.5)	61 (38.1)	.01
Peptic ulcer disease	6 (8.2)	20 (12.5)	.38
Cirrhosis	4 (5.5)	10 (6.3)	> .99
Hemiplegia	4 (5.5)	3 (1.9)	.21
Kidney disease	10 (13.7)	45 (28.1)	.02
Brittle diabetes mellitus	4 (5.5)	26 (16.3)	.02
Cancer	28 (38.4)	49 (30.6)	.29
Leukemia	1 (1.4)	9 (5.6)	.18
Lymphoma	2 (2.7)	8 (5.0)	.73
Moderate or severe liver disease	3 (4.1)	5 (3.1)	.71
Metastatic cancer	7 (9.6)	14 (8.8)	.81
Any inotropic agent, No. (%)	4 (5.5)	28 (17.5)	.01
Any vasoactive agent, No. (%)	32 (43.8)	108 (67.5)	.01
Fluid balance, median (IQR), mL	4945 (2019–8282)	6948 (3658–10,810)	.01

*APACHE* acute physiology, age, chronic health evaluation, *MAP* mean arterial pressure, *SOFA* sequential organ failure assessment

^a^From noninvasive blood pressure monitoring

^b^Seventh hour of early goal-directed therapy

^c^Defined as pre-admission minus post-resuscitation MAP

Regression analysis of ΔMAP as a continuous variable was not significantly associated with the rate of AKI (OR 0.99 [95% CI 0.97–1.01]; *p* = .31). The relationship of ΔMAP and AKI did not become significant with the addition of fluid balance to the model. Regression analysis of post-resuscitation MAP ≥ 65 mmHg was not significantly associated with AKI (OR 0.9 [95% CI 0.27–3.74]). When patients were categorized into quartiles on the basis of ΔMAP values (first quartile, − 24.5 to 3.9 mmHg; second quartile, 4.0–12.4 mmHg; third quartile, 12.5–19.9 mmHg; fourth quartile, 20.0–43.8 mmHg), in terms of age, sex, BMI, history of hypertension, CCI, APACHE III score, SOFA score, and fluid balance, patients in quartile 1 were similar to patients in quartiles 2 through 4. Patients in the first quartile had a lower percentage and odds of AKI compared with the other the third quartiles ([56.9% vs. 72.6%, respectively; *p* = .03] and [OR 0.50, 95% CI 0.27–0.93], respectively) (Additional file 1: Fig. S1 and Additional file 2: Fig. S2). Patients in the first quartile had a lower pre- (*p* < .01) and higher post-resuscitation MAP (*p* < .01) (Table 2). Rates of AKI for patients in second, third and fourth quartiles (*p* = .11, *p* = .51, *p* = .87, respectively) were not significantly different when compared with the rest of the cohort. In addition, following adjusting for history of hypertension, being within the first quartile was significantly associated with a lower rate of AKI (*p* = .04).

**Table 2:** Comparison of patients in quartile 1 versus quartiles 2–4 of delta mean arterial pressure (*N* = 233)

Characteristic	Quartile 1 (*n* = 58)	Quartiles 2–4 (*n* = 175)	*p* value
Age, median (IQR), year	66.3 (55.7–79.9)	72.1 (59.4–81.7)	.60
Body mass index, median (IQR), kg/m^2^	27.8 (23.7–32.8)	26.8 (22.2–31.6)	.23
Male sex, No. (%)	32 (55.2)	96 (54.9)	> .99
Pre-admission MAP, median (IQR), mmHg^a^	73.9 (67.8–81.9)	82.0 (76.3–91.0)	.01
Post-resuscitation MAP, median (IQR), mmHg^b^	79.0 (70.5–87.1)	66.0 (61.0–71.0)	.01
Acute kidney injury, No. (%)	33 (56.9)	127 (72.6)	.03
Stage 1	13 (17.8)	60 (82.2)	
Stage 2	14 (26.0)	40 (74.1)	
Stage 3	6 (18.2)	27 (81.8)	
Charlson comorbidity index, median (IQR)	7 (4.8–10.3)	7 (5.0–10.0)	.55
APACHE III score, 24 h, median (IQR)	78 (64.8–97.3)	82 (66–97)	.66
SOFA score, day 1, median (IQR)	6 (4–9)	7 (4–10)	.76
Culture positive septic shock (%)	29 (50)	87 (50)	.96
Positive culture source			.5
Blood (%)	5 (9)	22 (13)	
Urine (%)	5 (9)	19 (11)	
Respiratory (%)	16 (28)	33 (19)	
Wound (%)	2 (3)	5 (3)	
Other (%)	1 (1)	8 (4)	
Culture negative septic shock (%)	29 (50)	88 (50)	.96
Preexisting condition, No. (%)			
Hypertension	36 (62.1)	117 (66.9)	.53
Myocardial infarction	11 (19.0)	38 (21.7)	.71
Congestive heart failure	16 (27.6)	29 (16.6)	.08
Peripheral vascular disease	5 (8.6)	23 (13.1)	.49
Dementia	0 (0)	12 (6.9)	.04
Cerebrovascular accident	9 (15.5)	31 (17.7)	.84
Chronic pulmonary disease	16 (27.6)	54 (30.9)	.74
Rheumatic heart disease	7 (12.1)	16 (9.1)	.61
Diabetes mellitus	22 (37.9)	54 (30.9)	.34
Peptic ulcer disease	7 (12.1)	19 (10.9)	.81
Cirrhosis	2 (3.4)	12 (6.9)	.53
Hemiplegia	5 (8.6)	2 (1.1)	.01
Kidney disease	14 (24.1)	41 (23.4)	> .99
Brittle diabetes mellitus	7 (12.1)	23 (13.1)	> .99
Cancer	19 (32.8)	58 (33.1)	> .99
Leukemia	3 (5.2)	7 (4.0)	.71
Lymphoma	7 (12.1)	3 (1.7)	.01
Moderate or severe liver disease	2 (3.4)	6 (3.4)	> .99
Metastatic cancer	6 (10.3)	15 (8.6)	.79
Any inotropic agent, No. (%)	5 (8.6)	27 (15.4)	.27
Any vasoactive agent, No. (%)	35 (60.3)	105 (60.0)	> .99
Fluid balance, median (IQR), mL	6405 (2141–9723)	6102 (3210–9742)	.31

*APACHE* acute physiology, age, chronic health evaluation, *MAP* mean arterial pressure, *SOFA* Sequential organ failure assessment

^a^From noninvasive blood pressure monitoring

^b^Seventh hour of early goal-directed therapy

We used backward stepwise regression analysis of all relevant variables with 25% probability to enter and 10% probability to leave the model. The final model included being in the first quartile, CCI, APACHE III score, SOFA score, comorbidities, vasoactive agents, inotropic agents, pre-admission MAP, and post-resuscitation MAP. BMI, pre-admission MAP, inclusion in quartiles 2 through 4, and APACHE III scores were significant predictors of AKI, and these were entered into a nominal logistic regression analysis (Table 3). Compared with patients in quartiles 2 through 4 combined, those in quartile 1 had decreased odds of AKI (OR 0.26 [95% CI 0.11–0.57]) in a multivariate analysis controlling for all significant predictors. The c-statistic of this model was 0.80 for AKI, and Hosmer–Lemeshow test for goodness of fit showed excellent calibration (Chi-squared 3.6 and *p* value 0.9). When this same nominal logistic regression analysis was run with the addition of the history of hypertension variable, all four variables listed in Table 3 remained significant, and history of hypertension was not a significant predictor of AKI (*p* = 0.28).

**Table 3:** Regression model for prediction of acute kidney injury for all patients

Characteristic	Odds ratio	95% confidence interval	*p* value
Being in the first quartile^a^	0.26	0.11–0.57	.01
Body mass index	0.92	0.87–0.96	.01
APACHE III score, 24 h	0.96	0.94–0.97	.01
Pre-admission MAP	1.04	1.01–1.08	.01

*APACHE* acute physiology, age, chronic health evaluation, *MAP* mean arterial pressure

^a^Patients had ΔMAP values in the lowest quartile

### Hypertension subgroup analysis

One hundred fifty-three patients (66.0%) had a history of high blood pressure. When comparing those who did and did not have a history of hypertension, age (*p* = .01), BMI (*p* = .01), incidence of AKI (75.2% and 56.3%, respectively; *p* = .01), CCI (*p* = .01), and APACHE III score (*p* = .01) were significantly different (Table 4).

**Table 4:** Patient characteristics, stratified by history of hypertension (*N* = 233)

Characteristic	No hypertension (*n* = 80)	History of hypertension (*n* = 153)	*p* value
Age, median (IQR), year	62.5 (50.6–78.0)	74.5 (63.4–82.6)	.01
Body mass index, median (IQR), kg/m^2^	25.0 (21.2–28.7)	28.0 (23.3–33.6)	.01
Male sex, No. (%)	45 (56.3)	83 (54.2)	.78
Pre-admission MAP, median (IQR), mmHg^a^	77.3 (72.1–87.3)	81.3 (75.0–90.5)	.13
Post-resuscitation MAP, median (IQR), mmHg^b^	68.0 (62.2–76.5)	68.0 (62.0–74.4)	.98
ΔMAP, median (IQR), mmHg^c^	11.3 (3.0–17.9)	12.7 (4.0–21.0)	.18
Acute kidney injury, No. (%)	45 (56.3)	115 (75.2)	.01
Stage 1	25 (34.3)	48 (65.8)	
Stage 2	12 (22.2)	42 (77.8)	
Stage 3	8 (24.2)	25 (75.8)	
Charlson comorbidity index, median (IQR)	5 (3–7)	8 (6–11)	.01
APACHE III score, 24 h, median (IQR)	71 (61.3–88.8)	86 (69.5–103)	.01
SOFA score, day 1, median (IQR)	6 (4–9)	7 (4–10)	.40
Any inotropic agent, No. (%)	8 (10.0)	24 (15.7)	.32
Any vasoactive agent, No. (%)	46 (57.5)	94 (61.4)	.58
Fluid balance, median (IQR), mL	6419 (3334–8953)	6073 (2398–10,225)	.43

*APACHE* acute physiology, age, chronic health evaluation, *MAP* mean arterial pressure, *SOFA* Sequential organ failure assessment

^a^From noninvasive blood pressure monitoring

^b^Seventh hour of early goal-directed therapy

^c^Defined as pre-admission minus post-resuscitation MAP

In the subgroup analysis of patients with a history of hypertension, the ΔMAP values that fell into each quartile did not change significantly from the entire cohort (as described above). The ΔMAP in the first quartile ranged from −24.5 to 4 mmHg; quartile 2, from 4.1 to 12.7 mmHg; quartile 3, from 13 to 21 mmHg; and quartile 4, from 21.1 to 43.8 mmHg. Patients in the first quartile had similar risk factors for AKI as those in quartiles 2 through 4. AKI was less common for patients in the first quartile than the other 3 quartiles combined (62% vs. 80%, respectively; *p* = .03) (Table 5). Similarly, the odds of AKI was lowest for those in quartile 1 vs. quartiles 2 through 4 (OR 0.40 [95% CI 0.18–0.90]).

**Table 5:** Comparison of patients with hypertension in quartile 1 versus quartiles 2–4 of delta mean arterial pressure (*n* = 153)

Characteristic	First quartile (*n* = 39)	Second to fourth quartiles (*n* = 114)	*p* value
Age, median (IQR), year	73.7 (55.9–80.7)	74.8 (64.2–83.1)	.14
Body mass index, median (IQR), kg/m^2^	28.6 (23.9–34.1)	27.9 (23.3–33.6)	.39
Male sex, No. (%)	22 (56.4)	61 (53.5)	.85
Pre-admission MAP, median (IQR), mmHg^a^	75.0 (69.0–84.0)	83.5 (76.7–91.3)	.01
Post-resuscitation MAP, median (IQR), mmHg^b^	79.0 (71.5–86.0)	65.0 (61.0–70.3)	.01
Acute kidney injury, No. (%)	24 (61.5)	91 (79.8)	.03
Stage 1	9 (18.8)	39 (81.3)	
Stage 2	10 (23.8)	32 (76.2)	
Stage 3	5 (20.0)	20 (80.0)	
Charlson comorbidity index, median (IQR)	9 (6–12)	7.5 (6–11)	.62
APACHE III score, day 1, median (IQR)	88 (72–99)	85.5 (68.8–104.3)	.51
SOFA score, day 1, median (IQR)	6 (4–8)	7 (4–10)	.28
Preexisting condition, No. (%)			
Myocardial infarction	10 (25.6)	31 (27.2)	> .99
Congestive heart failure	14 (35.9)	25 (21.9)	.09
Peripheral vascular disease	5 (12.8)	22 (19.3)	.47
Dementia	0 (0)	10 (8.8)	.07
Cerebrovascular accident	8 (20.5)	28 (24.6)	.67
Chronic pulmonary disease	12 (30.8)	40 (35.1)	.70
Rheumatic heart disease	6 (15.4)	10 (8.8)	.24
Diabetes mellitus	18 (46.2)	48 (42.1)	.71
Peptic ulcer disease	6 (15.4)	18 (15.8)	> .99
Cirrhosis	0 (0)	8 (7.0)	.20
Hemiplegia	5 (12.8)	2 (1.8)	.01
Kidney disease	13 (33.3)	35 (30.7)	.84
Brittle diabetes mellitus	7 (17.9)	20 (17.5)	> .99
Cancer	15 (38.5)	36 (31.6)	.44
Leukemia	2 (5.1)	4 (3.5)	.65
Lymphoma	4 (10.3)	3 (2.6)	.07
Moderate or severe liver disease	0 (0)	4 (3.5)	.57
Metastatic cancer	5 (12.8)	10 (8.8)	.53
Any inotropic agent, No. (%)	3 (7.7)	21 (18.4)	.13
Any vasoactive agent, No. (%)	24 (61.5)	70 (61.4)	> .99
Fluid balance, median (IQR), mL	7193 (1349–10,280)	5733 (2820–10,220)	.32

ΔMAP is defined as pre-admission minus post-resuscitation MAP

*APACHE* acute physiology, age, chronic health evaluation, *MAP* mean arterial pressure, *SOFA* sequential organ failure assessment

^a^From noninvasive blood pressure monitoring

^b^Seventh hour of early goal-directed therapy

In the subgroup analysis of patients with a history of hypertension, backward stepwise regression analysis with all variables entered, as described above, showed that BMI, pre-admission MAP, APACHE III score, the presence of brittle diabetes mellitus, inclusion in quartiles 2 through 4, and norepinephrine use were statistically significant predictors of AKI (Table 6). Compared with the patients in quartiles 2 through 4 combined, patients in quartile 1 had lower odds of AKI (OR 0.12 [95% CI 0.04–0.37]) in multivariate analysis after controlling for significant predictors (Table 6). The c-statistic for this model of AKI was 0.87.

**Table 6:** Regression model for prediction of acute kidney injury for all patients with hypertension history

Characteristic	Odds ratio	95% confidence interval	*p* value
Being in the first quartile^a^	0.12	0.04–0.37	.01
Body mass index	0.90	0.84–0.96	.01
APACHE III score, 24 h	0.97	0.95–0.99	.01
Pre-admission MAP	1.07	1.01–1.12	.01
Brittle diabetes mellitus	6.03	1.36–44.19	.04
Norepinephrine use	3.72	1.42–10.54	.01

*APACHE* acute physiology, age, chronic health evaluation, *MAP* mean arterial pressure

^a^Patients had ΔMAP values in the lowest quartile

## Discussion

In this report, we identified a hemodynamic variable that was associated with AKI among patients with septic shock. When stratifying patients by ΔMAP, those in the first quartile of MAP change (−24.5 to 3.9 mmHg, i.e., patients with post-resuscitation MAP higher than or equal to their pre-admission MAP) had a significantly lower incidence of AKI. Being in the second to fourth delta-MAP quartiles, body mass index, APACHE III score, and pre-admission MAP were independently associated with risk of AKI.

These results suggest that having a ΔMAP value within the first quartile could be a modifiable risk factor that is associated with a lower risk of AKI in patients with severe sepsis and septic shock. Thus, pre-admission MAP values could be used to guide post-resuscitation MAP targets. In other words, our report suggests that a post-resuscitation MAP that is no more than 4 mmHg lower than pre-admission MAP may be protective against the development of AKI in this cohort. This guideline could potentially provide a specific, individualized MAP target for each patient with severe sepsis or septic shock. While our results provide a new hypothesis, the lack of a clear dose-response relationship between ΔMAP quartiles 2–4 and AKI could mean another variable plays a role in the development of SA-AKI. In either case, this deserves further attention in terms of prospective study.

The concept of ΔMAP itself is not quite novel. ΔMAP previously was investigated in high-risk patients undergoing cardiopulmonary bypass [18]. The authors reported a higher rate of AKI when MAP was at least 26 mmHg lower than baseline during cardiopulmonary bypass. We note that the ΔMAP targets described in that study specifically pertained to patients undergoing cardiac surgery and is not applicable to SA-AKI.

In a previous study of SA-AKI, Badin et al. [11] found that the time-averaged MAP in the early phase of acute circulatory failure was lower in patients who had septic shock, prior renal function impairment, and AKI. Our results confirm those of Badin et al. and go a step further; the decrement of post-resuscitation MAP relative to pre-admission MAP seems to be associated with the incidence of AKI. Having ΔMAP values in quartile 1 (i.e., patients with post-resuscitation MAP mostly higher than their pre-admission MAP) was significantly associated with a lower incidence of AKI for the total cohort. Additionally, the association of ΔMAP values in quartile 1 was independent of a history of hypertension. Our results further suggest that ΔMAP values may help better define an individualized goal MAP for each patient by taking into consideration pre-admission blood pressures and preexisting conditions such as chronic hypertension. Our results are similar to those of the SEPSISPAM (Sepsis and Mean Arterial Pressure) investigators’ trial [4], which reported an association between patients with a history of hypertension and a need for a higher goal MAP.

In our study, the non-AKI group had a median (IQR) post-resuscitation MAP of 71 (66–79) mmHg, whereas, among the AKI group, it was 66 (61–73) mmHg. These values were consistent with the findings of other reports [11, 12]. The mean post-resuscitation MAP of the group without AKI was lower than that suggested by the FINNAKI (Finnish Acute Kidney Injury) study [13]. Although Asfar et al. [4] proposed a lower MAP to avoid adverse effects of resuscitation, their low-target MAP group had an actual MAP of 75 mmHg, which is similar to our non-AKI group’s post-resuscitation MAP and the findings of other studies [4, 11, 13].

Our study has some limitations, including its retrospective and observational nature and that it was restricted to a single center. We could not control for interventions, including patients’ resuscitation MAP goals. While our investigation focused on the association of ΔMAP with an important renal outcome, effects on other organ systems were not included in this investigation and are unknown. The retrospective collection of noninvasive blood pressure readings from the electronic health record was not ideal since these blood pressures are collected in the hospital setting and may not reflect patients “healthy” blood pressure; we attempted to mitigate this problem by using the median of MAPs to prevent an outlier from skewing the patients’ pre-admission MAP values. As the main focus of this retrospective analysis was generating hypothesis for targeted AKI preventive interventions for future prospective study, we did not report the impact of delta-MAP on other organs including the cardiovascular system. Further, a retrospective collection of noninvasive blood pressure readings may have limited the number of patients in our analysis and may have inadvertently included more patients with preexisting hypertension or other comorbidities associated with regular blood pressure monitoring. We corrected for the higher prevalence of hypertension by including it in our multivariate model and confirming that it does not account for our findings. Indeed, our results were independent of a history of hypertension. Due to these limitations, this study may have limited generalizability. The strengths of this study include the detailed characterization of enrolled patients.

## Conclusions

Our study is the first to analyze ΔMAP, defined as pre-admission MAP minus post-resuscitation MAP, as a risk factor for SA-AKI. Patients with ΔMAP values in the lowest quartile (i.e., patients with post-resuscitation MAP mostly higher than their pre-admission MAP) had a significantly lower incidence of AKI independent of a history of hypertension. Analysis of the subgroup of patients with hypertension showed the same relationship. Our results are hypothesis generating and suggest that, for patients with severe sepsis or septic shock, clinicians may better define an individualized target MAP for the resuscitation phase goals by considering the patient’s pre-admission MAP. We recommend future studies to further explore the usefulness of this hemodynamic target for sepsis resuscitation.

## Additional files

**Additional file 1: Fig. S1.** Quartile of ΔMAP and Incidence of AKI (p-value = .03). Abbreviations: MAP, mean arterial pressure; AKI, acute kidney injury.

**Additional file 2: Fig. S2.** Quartile of ΔMAP and highest stage of severity of AKI during hospitalization (p-value = .03). Abbreviations: MAP, mean arterial pressure; AKI, acute kidney injury.

## Abbreviations

AKI: acute kidney injury
APACHE: acute physiology, age, chronic health evaluation
BMI: body mass index
CCI: Charlson comorbidity index
EGDT: early goal-directed therapy
ICU: intensive care unit
IQR: interquartile range
MAP: mean arterial pressure
OR: odds ratio
SA-AKI: sepsis-associated acute kidney injury
sCR: serum creatinine
SOFA: sequential organ failure assessment
UOP: urine output
