# Supplementary figures and images for: Impact of individualized target mean arterial pressure for septic shock resuscitation on the incidence of acute kidney injury: a retrospective cohort study
Source: Ann Intensive Care. 2018 Dec 10;8:124. doi: 10.1186/s13613-018-0468-5 (PMC6288098; doi:10.1186/s13613-018-0468-5)

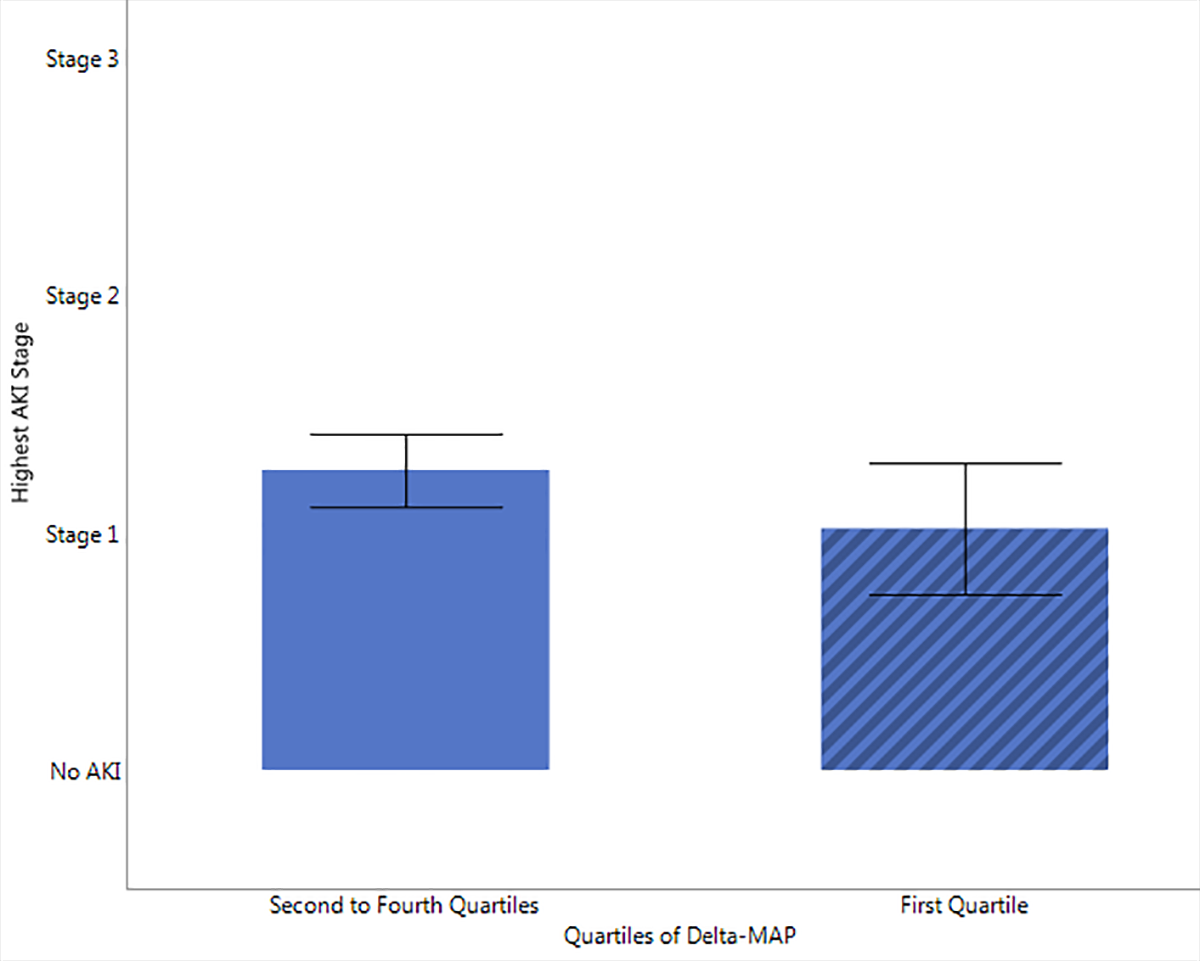

Supplement: Supplementary file 1 — Additional file 1: Fig. S1. Quartile of ΔMAP and Incidence of AKI (p-value = .03). Abbreviations: MAP, mean arterial pressure; AKI, acute kidney injury. [file 13613_2018_468_MOESM1_ESM.tif]

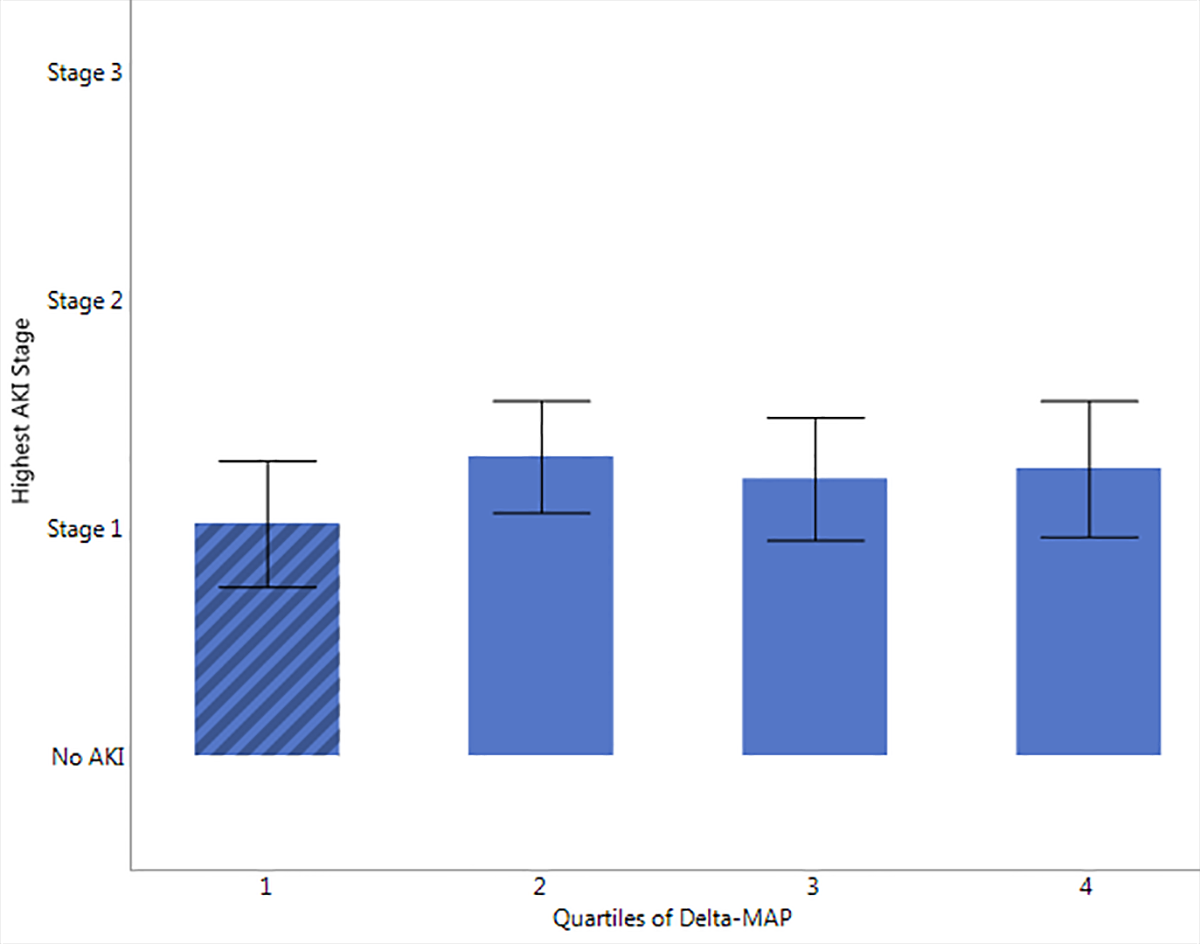

Supplement: Supplementary file 2 — Additional file 2: Fig. S2. Quartile of ΔMAP and highest stage of severity of AKI during hospitalization (p-value = .03). Abbreviations: MAP, mean arterial pressure; AKI, acute kidney injury. [file 13613_2018_468_MOESM2_ESM.tif]
